# Preferences for interventions designed to increase cervical screening uptake in non‐attending young women: How findings from a discrete choice experiment compare with observed behaviours in a trial

**DOI:** 10.1111/hex.12992

**Published:** 2019-10-28

**Authors:** Helen E. Campbell, Alastair M. Gray, Judith Watson, Cath Jackson, Carly Moseley, Margaret E. Cruickshank, Henry C. Kitchener, Oliver Rivero‐Arias

**Affiliations:** ^1^ National Perinatal Epidemiology Unit Nuffield Department of Population Health University of Oxford Oxford UK; ^2^ Health Economics Research Centre Nuffield Department of Population Health University of Oxford Oxford UK; ^3^ Department of Health Sciences University of York York UK; ^4^ Institute of Cancer Sciences The University of Manchester St Mary’s Hospital Manchester UK; ^5^ University of Aberdeen Aberdeen Centre for Women’s Health Research Aberdeen UK

**Keywords:** cervical cancer, discrete choice experiments, heterogeneity, preferences, screening uptake, United Kingdom, young women

## Abstract

**Background:**

Young women’s attendance at cervical screening in the UK is continuing to fall, and the incidence of invasive cervical cancer is rising.

**Objectives:**

We assessed the preferences of non‐attending young women for alternative ways of delivering cervical screening.

**Design:**

Postal discrete choice experiment (DCE) conducted during the STRATEGIC study of interventions for increasing cervical screening uptake. Attributes included action required to arrange a test, location of the test, availability of a nurse navigator and cost to the National Health Service.

**Setting and participants:**

Non‐attending young women in two UK regions.

**Main outcome measures:**

Responses were analysed using a mixed multinomial logit model. A predictive analysis identified the most preferable strategy compared to current screening. Preferences from the DCE were compared with observed behaviours during the STRATEGIC trial.

**Results:**

The DCE response rate was 5.5% (222/4000), and 94% of respondents agreed screening is important. Preference heterogeneity existed around attributes with strong evidence for test location. Relative to current screening, unsolicited self‐sampling kits for home use appeared most preferable. The STRATEGIC trial showed this same intervention to be most effective although many women who received it and were screened, attended for conventional cytology instead.

**Conclusions:**

The DCE and trial identified the unsolicited self‐sampling kit as the most preferred/effective intervention. The DCE suggested that the decision of some women receiving the kit in the trial to attend for conventional cytology may be due to anxieties around home testing coupled with a knowledge that ignoring the kit could potentially have life‐changing consequences.

## 1 INTRODUCTION

For a growing proportion of young women, embarrassment, anxiety, fear and inconvenience around testing are barriers to cervical screening.^1^, ^2^, ^3^, ^4^ The reluctance of many to engage with the National Health Service (NHS) Cervical Screening Programme (CSP) is reflected in national screening statistics. Five‐year screening coverage rates in England are lowest amongst 25‐ to 29‐year‐old women and are falling (72% in 2005 to 65% in 2017), whilst the incidence of invasive cervical cancer has begun to increase.^5^, ^6^ Similarly in Scotland, non‐attendance is highest amongst young women, with a screening uptake rate during 2017/2018 of just 62% in those aged 25 to 29 years.^7^ Furthermore, screening uptake is known to be lower with increasing levels of deprivation, with uptake rates amongst young women shown to vary as much as 4% between highest and lowest deprivation categories.^8^, ^9^ This is a concern and is driving research into tailored interventions aiming to address women’s barriers to screening and which could potentially be embedded within the existing CSP to increase uptake.

The decision to invest in any such interventions should be informed by evidence on effectiveness and cost‐effectiveness. Complementary data on the acceptability of an intervention to women might also reassure policymakers that the intervention will work in routine practice. Discrete choice experiments (DCEs) offer a way of eliciting preferences for the characteristics or attributes of an intervention; they also provide information on the trade‐offs (the decision to sacrifice the benefits of one attribute in favour of another) that respondents are prepared to make between attributes. DCEs have been used in health‐care settings to improve the design and delivery of patient services.^10^, ^11^, ^12^ Their use also extends to cancer screening programmes; a systematic review identified 22 stated preference studies published between 1990 and 2013 addressing breast, cervical and colorectal cancer screening.^13^

The DCE reported in this paper was conducted as part of the STRATEGIC study (Strategies to Increase Cervical Screening Uptake at First Invitation) which evaluated novel interventions intended to make screening more acceptable to young women receiving their first invitation.^14^, ^15^ Interventions were evaluated in a phased cluster randomized trial conducted in the Greater Manchester area of England and the Grampian area of Scotland. In phase 1, pre‐invitation information leaflets and Internet booking options were trialled to increase initial attendance. In phase 2, young women who had not attended within six months of an initial invitation were randomized to one of six alternatives: a human papillomavirus (HPV) self‐sampling kit (SSK) for home use which was trialled as an unsolicited (sent) and a solicited (offered) intervention, a pre‐specified timed appointment (PTA) for a routine screening test, access to a nurse navigator (NN) whom women could contact to discuss concerns about screening and for help in navigating the screening process, a choice between a PTA and the NN, or no further action. The feasibility of these interventions was confirmed in a pilot study. The primary outcome measure for phase 2 of the trial was uptake in screening at 12 months post‐initial invitation. Cost‐effectiveness was also assessed.^15^, ^16^

The DCE was conducted simultaneously with the trial. Its objectives were to determine young women’s preferences for the individual characteristics of the phase 2 interventions and to predict which interventions were likely to be most preferable to women. The study design also afforded a unique opportunity to compare the effectiveness of the STRATEGIC interventions with their potential acceptability to women if implemented widely in the UK. Given the defining characteristics of the study cohort (a hard to reach, growing group of young women about whom little is known regarding their preferences for screening interventions), this is a novel study which should be of interest to policymakers, clinicians, researchers and women themselves.

## 2 METHODS

### 2.1 Designing the discrete choice experiment

In a DCE, participants are presented with a number of questions, each asking them to make a choice between two or more alternatives (together referred to as a choice set). The alternatives within a choice set are described using a set of common attributes (eg in a study of follow‐up care, attributes might include frequency of follow‐up, clinical personnel seen and duration of appointment).^10^ Each attribute has values (levels), and the levels taken by at least one attribute are systematically varied between the alternatives (eg follow‐up frequency might be annual in one alternative and 2 yearly in the other). Respondents then select their preferred alternative.^17^ Here, the alternatives considered were different means of delivering the CSP (see Table 1). The DCE was developed taking the following steps.

**Table 1:** Characterization of phase 2 STRATEGIC interventions through combinations of attributes (and levels) incorporating issues of known importance to non‐attending women

Attributes and levels	Interventions			
	PTA	Sent HPV SSK	Offered HPV SSK	NN
Action required by you personally to arrange a test?				
Yes			●	●
No	●	●		
Location of the test				
GP surgery/clinic	●			●^a^
Home		●	●	●^b^
Nurse available for discussion or help prior to appointment?				
Yes				●
No	●	●	●	
Cost of your test to the NHS				
£8		●	●	
£20				●^b^
£25	●			
£40				●^a^

Abbreviations: HPV SSK, self‐sampling kit; NN, nurse navigator; PTA, pre‐specified timed appointment.

a NN plus clinic test.

b NN plus HPV SSK.

### 2.2 Identifying attributes and levels

The published evidence around barriers to cervical screening helped develop a topic guide for planned qualitative interviews with 20‐30 women.^3^, ^4^ Interview invitations were sent to a sample of women invited to take part in phase 1 of STRATEGIC and to women in the phase 2 pilot study. It was the intention to use purposive sampling so as to conduct interviews in a mix of women offered the phase 2 interventions (HPV SSK, PTA, NN), and furthermore in those who had accepted and declined the intervention. A first batch of 600 invitations yielded four respondents; a second batch of 500 yielded one additional respondent. These five women underwent semi‐structured telephone interviews. All had been allocated the sent HPV SSK but prior to interview it was not known if they had used the kit. Interviews were digitally recorded, transcribed verbatim and analysed using deductive content analysis.^18^

For the women interviewed, four issues emerged as being important. Flexibility, particularly around the booking process and attending for the screen (fitting this into busy daily life), was a key factor. Expertise was also important, with regard to self‐testing versus testing by a health professional. The emotional impact of screening was evident, centring upon the discomfort of testing (regardless of location), and the embarrassment of having a test administered by a health professional. Finally, women raised ideas around greater normalization of screening, including the provision of more information of the process, as well as increased use of reminders (delivered, eg by text message).

As only five women were interviewed, findings were reviewed against published evidence on the barriers to cervical screening and found to be broadly consistent. Together, STRATEGIC’s clinical, quantitative and qualitative teams ensured the finalized list of attributes captured aspects of screening that were important to women and also described features of the phase 2 interventions.

Table 1 shows these attributes, their levels, and how each relates to the interventions being studied. Inconvenience around the booking process was a concern, as was a dislike of the test per se, which potentially makes calling for an appointment difficult. As the phase 2 interventions offered different approaches for arranging a screen (eg with a PTA the appointment is made for the woman), we included the attribute ‘Action required by you personally to arrange a test?’ with associated levels ‘Yes’ and ‘No’. The inconvenience of attending, coupled with the embarrassment of a gynaecological examination, could potentially be overcome by the use of a HPV SSK. Some women, however, have concerns around their competence to perform a home test.^19^ To account for these aspects, we included the attribute ‘Location of the test’ with levels ‘GP surgery/clinic’ and ‘Home’. Women’s perceived need for more information plus their fears around the examination could be allayed by a trained nurse prior to screening. As the NN could potentially be an adjunctive option with any of the interventions, it was included as an attribute in its own right. Finally, a cost attribute was included with four values reflecting the approximate costs to the NHS of each of the interventions.

### 2.3 Experimental design

The attributes and levels in Table 1 combined to give 2 × 2 × 2 × 4 = 32 possible profiles and 1024 (32^2^) possible pairwise choice tasks. To reduce the number of choice tasks, a D‐optimal design approach in Ngene 1.1.2 software was used.^20^, ^21^ This method ensures that choice sets are chosen to provide a balance of attribute levels across the experiment (orthogonality), and that attributes within a choice set never take the same level value (thereby forcing respondents to trade on all attributes and eliciting maximum information).^22^, ^23^, ^24^ The design approach suggested little informational benefit would be gained by using more than 16 choice sets, but given the challenges engaging these women with the qualitative interviews, we opted for 12 sets to reduce respondent burden (see Supplemental File 1).

### 2.4 Piloting of the questionnaire

As well as the choice sets, women completing the questionnaire reported their general views on the current screening programme and provided demographic information. In addition to the paper‐based questionnaire, an online version was developed using LimeSurvey (http://www.limesurvey.org). The weblink was sent out with the paper‐based questionnaire, thus providing women with a choice as to how to respond. To encourage participation, women were offered a £10 high‐street voucher.

Piloting of the questionnaire took place between July and September 2014 in young women not responding to their initial screening invitation. The English and Scottish screening agencies identified eligible women and mailed out the questionnaires. One thousand questionnaires were sent to women in Greater Manchester (n = 650) and Grampian (n = 350), and 54 (5.4%) were returned (11/54 (20%) using the online version). Responding women appeared to complete the questionnaire without difficulty, and in response to a small number reporting, it was unclear when the NN would be available, the words ‘prior to the appointment’ (Table 1) were added. A graphic design team improved the look of the questionnaire, the final version of which and the accompanying information leaflet are shown in the Supplemental File 1.

### 2.5 Sample size

Sample size calculations for DCEs identify the minimum number of choice observations required to obtain reliable parameter estimates from the statistical models used to analyse stated choice data. In this study, for example, one completed questionnaire provided 12 choice observations. Applying the approach of Rose and Bliemer to response data from the pilot study showed that with sample sizes >150, it would be possible to estimate significant coefficients for the action, nurse and cost attributes. The location attribute would require a sample size of 1151 (see Supplemental File 1).^25^

### 2.6 Administering the final questionnaire

The main DCE data collection was carried out between mid‐July 2015 and mid‐September 2015. Three thousand questionnaires were sent to women in Greater Manchester (n = 2000) and Grampian (n = 1000). Questionnaires were again mailed out by the English and Scottish screening agencies, who in conjunction with the STRATEGIC study research team, identified young women in the Greater Manchester and Grampian regions of Scotland who had been invited for but who had not yet attended for their first cervical cancer screen. Young women, for whom a screening result had been recorded, were not eligible.

### 2.7 Statistical analysis

Descriptive statistics were used to summarize participant demographics and women’s views of screening. For the 12 choice sets, we assumed each choice was made in accordance with random utility theory which states that when faced with two or more alternatives, each of which generate a level of utility (satisfaction), an individual will select the alternative maximizing their utility.^17^ As researchers cannot observe an individual’s utility, but do have information on the attributes of the alternatives being considered, and of the decision makers per se, regression‐based modelling can be used to explain the statistical relationship between observed factors and the decision maker’s choices (more detail is provided in the Supplemental File 1). For this study, the explainable utility (V) of the *n*th individual for screening intervention (i) was estimated to be a linear and additive function of each intervention’s attributes and levels as follows:

(1) $$V_{\mathrm{ni}}=\alpha +\beta_{1}{Action}_{yes}+\beta_{2}{Location}_{GPsurg/clinic}+\beta_{3}{Nurse}_{yes}+\beta_{4}Cost$$

α is a constant term and if statistically significant suggests respondents have a general propensity to select alternative one over alternative two in choice sets, all else being equal. Its inclusion in the model allows this bias to be controlled for.^17^ As action, location and nurse are categorical variables, the sign of the estimated coefficients $\beta_{1}$, $\beta_{2}$ and $\beta_{3}$ denotes whether a move from the reference category for each variable (from no to yes for action, home to GP clinic for location and no to yes for nurse) results in an increase or decrease in overall utility. For cost, the directional change in utility resulting from an increase of £1 is given by $\beta_{4}$.

Equation 1 assumes the incremental impact of an attribute on utility is the same for all women. In reality, different forms of heterogeneity exist. It is possible to explain some of this heterogeneity by incorporating women’s characteristics in the explainable utility (V). However, a preferred alternative is to assume that there are different sources of heterogeneity that are not observable by the analyst and that are random. Accordingly, we estimated a random correlated parameter mixed logit model to account for this random heterogeneity (see Supplemental File 1). During the estimation process, parameter coefficients α, $\beta_{1}$, $\beta_{2}$ and $\beta_{3}$ were assumed to follow a normal distribution and $\beta_{4}$ (cost parameter) a lognormal distribution. Model results are presented as means and standard deviations for each parameter. Significant standard deviations indicate the presence of preference heterogeneity.

A predictive analysis was used to simulate hypothetical scenarios, each offering a dichotomous choice between the current CSP and a programme with attributes matching one of the STRATEGIC interventions. For each scenario, the probability that each alternative was chosen as best was predicted by the model after setting each of the attributes to levels characterizing the alternative programmes. The current CSP was defined as follows: action required to arrange a test? – ‘yes’, location of test? – ‘GP surgery/clinic’, availability of a nurse? – ‘no’ and cost of test to the NHS – ‘£25’. When simulating results for the PTA for example, only the action attribute was switched from yes to no. Confidence intervals around the predicted probabilities were estimated using simulation, in which for each model coefficient, 1000 draws were generated from a multivariate normal distribution with means and covariance matrix from the original correlated mixed logit model. Predicted probabilities were computed for each sampled coefficient and for each of the competing programmes, and then, 2.5th and 97.5th percentiles across 1000 predicted probabilities were identified.

Analyses were performed in Stata MP using the ‘mixlogit’ command.^26^, ^27^

### 2.8 The STRATEGIC trial results

In phase 2 of the STRATEGIC trial at 12 months post‐initial invitation, screening uptake in the no action (control) arm was 16.2%.^14^, ^15^ Only two of the interventions – the sent HPV SSK and the PTA – resulted in significantly higher 12‐month screening uptake rates of 21.3% and 19.8%, respectively (associated odds ratios 1.51 [95% CI 1.20‐1.91] and 1.41 [95% CI 1.14‐1.74]).^14^, ^15^ The NN resulted in a significant reduction in screening uptake. Of the women who received a sent HPV SSK and were screened, more than two thirds underwent only a conventional cytology test.^14^, ^15^

The accompanying cost‐effectiveness analysis concluded that the sent HPV SSK and PTAs were likely to be cost‐effective.^16^

## 3 RESULTS

**Figure 1:**
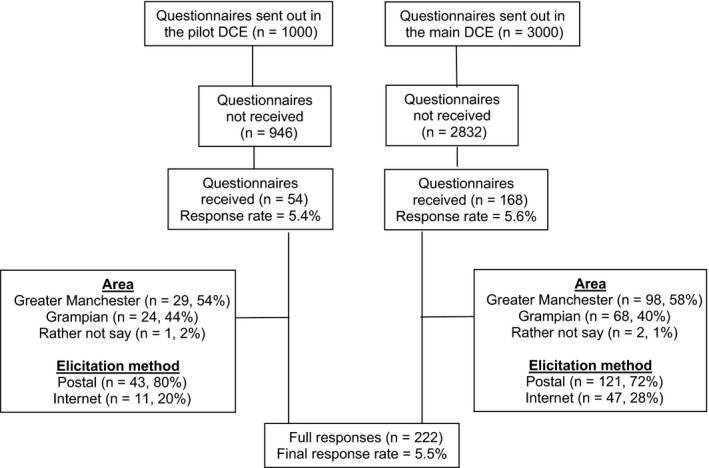
Flow of women through the study

Of the 3000 questionnaires sent, 168 (5.6%) were completed (98 [58%] from Greater Manchester, 68 [40%] from Grampian and with two women choosing not to say). One hundred and twenty‐one (72%) postal questionnaires were returned and 47 (28%) women completed the survey online. The low response rate and implications of the sample size for the statistical modelling were discussed. As changes to the wording of the questionnaire between the pilot and main surveys were minor and given the same experimental design was used, a pragmatic decision was made to combine the pilot and main surveys, giving a final sample of 222 respondents and 2600 choice observations to estimate reliable parameter estimates for all four attributes. Figure 1 details the flow of participants.

### 3.1 Descriptive data analysis

Table 2 shows respondents’ characteristics. Almost three fifths were from England and the overall mean age was 24.6 years. Women from Scotland were younger (reflecting the lower age threshold for commencement of screening in Scotland at the time). The majority of women were of White ethnicity and just under a half had been educated to university level. Almost three fifths were in employment.

**Table 2:** Characteristics of DCE respondents

Characteristics	N = 222
Country of residence – n (%)	
England	127 (57)
Scotland	92 (41)
I’d rather not say	3 (1)
Age in years – mean (SD)	24.6 (2.5)
England	26.4 (0.9)
Scotland	22.1 (1.8)
Missing	6
Ethnicity – n (%)	
White British/Irish	174 (79)
Any other White background	15 (7)
Pakistani	11 (5)
Other	9 (4)
Indian	4 (2)
African	4 (2)
Bangladeshi	1 (0)
I’d rather not say	1 (0)
Missing	3
Highest level of education – n (%)	
University	108 (49)
Further education to age 18 years	47 (21)
Vocational qualification	44 (20)
School leaver at age 16	13 (6)
I’d rather not say	10 (5)
Main activity – n (%)	
Employed	127 (57)
Student (full or part‐time)	57 (26)
Homemaker looking after the family	18 (8)
Unemployed and seeking work	7 (3)
I’d rather not say	5 (2)
Unpaid voluntary work	4 (2)
Long‐term sick or disabled	4 (2)

Abbreviation: SD, standard deviation.

When presented with the statement ‘screening for cervical cancer is important’, almost two thirds of women (64%) indicated that they strongly agreed and a further 30% selected the ‘agree’ option.

### 3.2 Discrete choice modelling analysis

Table 3 shows the mean coefficients for each attribute from the model. Each of the attributes had a significant impact upon women’s utility. The alternative specific constant was also significant (with a negative sign), suggesting that all else being equal, women selected the first of the two options presented. When considered in isolation, having to take action to arrange a test, having a test at the GP surgery/clinic and higher screening costs resulted in a decline in utility. The NN was associated with an increase in utility. Aside from the constant, the standard deviations for all coefficients were significant, suggesting considerable heterogeneity between women in their preferences. The standard deviation for the location coefficient was particularly large.

**Table 3:** Results from the final random correlated parameter mixed logit model

Means	Random correlated parameter mixed logit model^a^	
	Coefficient (SE)	*P* value
Constant	−0.431 (0.126)	.001
Action required by you?		
Yes	−0.521 (0.238)	.029
Location of test		
GP surgery/clinic	−1.123 (0.425)	.008
Nurse available for discussion or help prior to appointment?		
Yes	1.862 (0.336)	.000
Cost of your test to the NHS	−2.234 (0.140)	.000
**Standard deviations**	**Coefficient (SE)**	***P* value**
Constant	0.207 (0.158)	.190
Action required by you?		
Yes	2.279 (0.346)	.000
Location of test		
GP surgery/clinic	6.328 (0.761)	.000
Nurse available for discussion or help prior to appointment?		
Yes	2.695 (0.388)	.000
Cost of your test to the NHS	1.269 (0.116)	.000
**Goodness of fit**	**Value**	
Log‐likelihood	−958.19	
AIC/n	0.739	
Number of parameters	20	
Number of observations (n)	2647	
Number of women	222	

Abbreviations: AIC, Akaike’s information criteria; SE, standard error.

a An expanded version of these results including the 20‐parameter model is available from the authors on request.

Table 4 reports the predictive analysis. Women appeared indifferent between the current CSP and the use of PTAs; predicted probabilities of selecting each option were 0.503 and 0.497, respectively. Model predictions suggested both offered and sent SSKs were preferred to current screening practice. In contrast, current screening practice appeared preferable when compared with a programme in which a NN was offered as a standalone intervention.

**Table 4:** Comparison of predictions for each alternative proposed screening intervention with current screening practice

Choice between	Probability (95% CI)
Current practice^a^	.503 (.434 to .579)
Timed appointment^b^	.497 (.421 to .566)
Current practice^a^	.320 (.264 to .377)
Offered HPV SSK^c^	.680 (.623 to .736)
Current practice^a^	.299 (.250 to .357)
Sent HPV SSK^d^	.701 (.643 to .750)
Current practice^a^	.571 (.513 to .635)
Nurse navigator^e^	.429 (.365 to .487)

Abbreviation: HPV SSK, self‐sampling kit.

a Attribute levels for current practice are – action, yes; location, GP/clinic; nurse, no; cost £25.

b Attribute levels as for current practice with exception of action, which is switched from yes to no.

c Attribute levels as for current practice with exception of location, which is switched from GP/clinic to home, and cost, which is changed from £25 to £8.

d Attribute levels as for current practice with the exception of action, which is switched from yes to no, location which is switched from GP/clinic to home, and cost which is changed from £25 to £8.

e Attribute levels as for current practice with the exception of nurse, which is switched from no to yes, and cost which is changed from £25 to £40.

### 3.3 Comparing DCE and trial results

In the DCE predictive analysis, the sent HPV SSK was the most preferred alternative, which was also identified in the STRATEGIC trial as one of the two interventions potentially effective. The other intervention was the PTA, yet women in the DCE appeared indifferent between a PTA and the current CSP. The offered HPV SSK was preferred by women in the DCE but was not effective in the trial, and in both studies, the NN did not appear to be considered beneficial by women.

## 4 DISCUSSION

This DCE is the first to assess preferences for alternative means of delivering cervical screening in a group of young women reluctant to engage with the current NHS CSP. Additionally, the STRATEGIC study design afforded a unique opportunity to consider women’s stated preferences alongside observed behaviours in the trial, and so provides additional information to help interpret the trial findings.

The study was conducted in accordance with good practice guidance for DCEs.^28^, ^29^, ^30^ Qualitative interviews, the published literature and expert opinion were used to identify attributes. Additionally, a cost attribute was included to allow estimation of the marginal monetary benefit women placed upon each of the remaining attributes. For example, the monetary value of the benefit achieved when moving from a GP clinic setting to a home testing setting. However, as we were interested in women’s preferences for screening programmes as defined by combinations of attributes, we questioned the meaningfulness of these individual values and ultimately chose not to report them. Careful consideration was given to the experimental design in a bid to increase participation, the questionnaire was piloted, and the results used to inform sample size calculations for the main survey. Choice data were analysed using a discrete choice model accounting for different sources of random heterogeneity.

Almost 94% of women surveyed either strongly agreed or agreed with the statement ‘screening for cervical cancer is important’. This is a notable finding, which has been observed elsewhere. In conducting interviews with young non‐attending women for example, Waller et al found many were positively disposed towards cervical screening despite making a decision not to attend.^2^

The mean coefficients from the model (Table 3) showed that when holding all else constant, having to take action to arrange a test would result in a decline in utility when compared to not having to take action. Similarly, having a test at a GP surgery or clinic would result in lower utility than using a HPV SSK at home. When considered in isolation, a NN appeared to increase utility as indicated by the coefficient’s positive sign. These findings appear intuitive given what is known about women’s barriers to screening. There was considerable heterogeneity between women in terms of their preferences for the location of the test. This is perhaps not surprising as this attribute will capture dual (and opposing) concerns relating to embarrassment (whereby home testing is preferred) and expertise required to perform the test (whereby GP clinic‐based testing is preferred).

The ability to consider the DCE findings alongside the STRATEGIC trial results is a novel aspect of this work. In the DCE predictive analysis, women stated an obvious preference for an alternative exemplifying the use of a sent HPV SSK. The trial similarly showed sent HPV SSKs to be the most effective and cost‐effective intervention; however, it also unexpectedly revealed that many women screened after receiving a sent HPV SSK did not use the kit, but attended for conventional cytology screening.

A possible explanation is that there exists a sub‐group of women for whom home testing is not preferable. The DCE showed location was most important to women and revealed significant heterogeneity in preferences for this attribute. For some women, home may not be a safe or a private environment. Furthermore, research into women’s perspectives of HPV self‐testing has identified concerns including low confidence in the ability to self‐sample, worries that samples may be lost or contaminated in the post, that HPV tests may be inferior to cytology tests, and fears about a lack of professional input and low confidence in the test result.^19^ Here, it is possible that uncertainties around the HPV SSK *per se* exerted a greater influence on women’s screening decisions than the general process of how self‐sampling is delivered as covered by the DCE. Given concerns around HPV SSKs, but with an acceptance of the importance of screening, many women appeared to have been ‘nudged’ into attending for conventional cytology after receiving a kit at home.^14^

The trial also identified PTAs as a potentially effective option for increasing screening uptake, whereas women in the DCE appeared indifferent between routine screening appointments they make themselves and PTAs (Table 4). It is possible that differences exist between women’s preferences for hypothetical scenarios (in the DCE, women may have been communicating their general dislike of clinic‐based testing *per se*, regardless of how the appointment was arranged) and the decisions they face in reality; women may still dislike clinic‐based tests but are more likely to respond to a PTA because it removes the onus on them to arrange an appointment, and, as discussed above, they recognize that a decision not to attend could potentially have life‐changing consequences.

In the DCE, the offered HPV SSK was preferred to the current CSP, but was not effective in the trial. Again it is possible that whilst women in principle prefer the idea of home testing, in reality, with only an offer of a HPV SSK, the onus is still on them to request a kit. For many, this may still be a difficult first step to take.

Finally, the DCE suggested a screening programme incorporating a NN may be less preferable to women than the current CSP specification. Analogously, the trial showed the offer of a NN to have a detrimental impact upon screening uptake. As the trial investigators hypothesized the apparent need for a NN may have further heightened women’s anxieties and negative perceptions of screening.^14^, ^15^

Similar findings, where hypothetical choices differ from real choices, have been observed elsewhere and are considered to be due to hypothetical bias.^31^ This bias is consequence driven; in real scenarios, real consequences follow a decision and participants make choices in the knowledge of this. In DCEs, where participants face no consequences as a result of the choices they make, this is thought to alter and bias their responses.^31^ Considering this in the context of STRATEGIC, and in the case of PTAs, this would fit well with our hypotheses that whilst women in the DCE were indifferent between making or receiving an appointment, women receiving a PTA in the trial might possibly have considered the potential consequences of ignoring that appointment too great a risk, despite their dislike of cytology screening. We must, however, be cautious in drawing such conclusions, as the DCE could not fully depict all interventions as delivered in the trial. It was not possible to provide women in the DCE with detailed information about how to conduct and return a HPV SSK; rather, women were told only that they could undertake a similar test at home. The availability of differing amounts of information to women is also likely to have influenced their choices.

This study is not without limitations. In developing the attributes and levels, only five women were interviewed; however, drawing on the literature and expert views ensured confidence in the final set used for the DCE. The response rate of just 5.5% to the DCE is also a concern. Despite efforts to increase participation, the defining characteristic of this cohort of women, that is that they are non‐engaged, meant that engaging them with a DCE about a screening programme in which they have chosen not to participate was challenging. Other DCEs in the area of cervical screening have similarly reported low response rates in sub‐groups of women eligible for but not attending screening.^32^ As non‐responding women are likely to be the most at‐risk group, future studies may need to explore alternative ways of engaging such women to understand the issues they face with regard to cervical screening.

Given the low response rate, there is potential for non‐response error. However, it is difficult to know how we might have mitigated this further. Watson et al used meta‐regression to identify factors of DCEs influencing response rates, and reported more than 2‐4 attributes were associated with reduced response rates but failed to find a negative association between response rates and the number of attribute levels.^33^ The use of reminders increased response rates, but the way this survey had to be administered (the details of women were known only to the screening agencies) meant the research team were unable to monitor responses and send out reminders. The low response rate raises obvious questions about the generalizability of the study findings. Whilst our DCE results appear to be intuitive and commensurate with previous research on barriers to cervical screening, we must nevertheless exercise caution in drawing wider generalized conclusions from our work.

Finally, it is important to consider the implications of recent changes to the cervical screening environment for the findings reported here. HPV vaccination of girls aged 12 to 13, for example, has been offered by the NHS since 2008, and in September 2019 will be extended to boys of the same age.^34^ Studies have reported significant reductions in low‐ and high‐grade cervical intraepithelial neoplasia amongst those vaccinated, yet maintain that HPV vaccination in combination with regular cervical screening is the best way to prevent against cervical cancer, as vaccination does not protect against all types of cancer‐causing HPV.^35^ Research into the effectiveness and acceptability of interventions, which increase the likelihood of a young women attending for cervical screening, therefore, continues to be paramount.

## 5 CONCLUSIONS

This paper presents previously unreported data on the stated preferences of a group of hard to reach young women for the characteristics of interventions, which could potentially be embedded within the NHS CSP to increase screening uptake. In the DCE as well as the STRATEGIC trial, the sent SSK was the most preferred/effective alternative. Women in the DCE strongly acknowledged the importance of screening yet differed significantly in their preferences for the location of the test. This could offer a potential means of understanding why many women screened after receiving the sent HPV SSK in the trial attended for conventional cytology.

Policies bringing unsolicited screening interventions directly to women appear likely to be most effective. Additionally, such interventions might also cause women to contemplate the potential implications of not responding to the intervention (a PTA or HPV SSK) in front of them; however, this would need to be explored more fully by future research. The low DCE response rate and the potential implications for the generalizability of the results must be acknowledged. Future research in this area should consider whether such questionnaires, which may be intimidating for respondents, offer the best means of engaging with a hard to reach cohort of women.

## 6 COMPLIANCE WITH ETHICAL STANDARDS

STRATEGIC was granted ethical approval by NRES Committee North West – Greater Manchester North on 01/11/11 (REC reference 11/NW/0624). The DCE was included in the original protocol and in the ethical amendment number 5 (dated 11/09/12) with a further amendment (number 8) approved on 29/01/14. The study was conducted in accordance with the ethical principles outlined in the 1964 Declaration of Helsinki and its later amendments or comparable ethical standards. Informed consent was obtained from all individual participants included in the study.

## CONFLICT OF INTEREST

None.

## Data Availability

Data available on request from the authors.
