# Supplementary material for: Preferences for interventions designed to increase cervical screening uptake in non‐attending young women: How findings from a discrete choice experiment compare with observed behaviours in a trial
Source: Health Expect. 2019 Oct 28;23(1):202–11. doi: 10.1111/hex.12992 (PMC6978852; doi:10.1111/hex.12992)
Supplement: Supplementary file 1 [file HEX-23-202-s001.docx]

**Supplemental File 1: Preferences of non-attending young women for the characteristics of interventions to increase cervical cancer screening uptake – a discrete choice experiment**

**Experimental Design**

A D-optimal approach to design attempts to elicit maximum information for preference estimation.^1-3^ Values of D-optimality can be computed for DCE designs that use different numbers of choice tasks, with higher values of the statistic being preferable. Our calculations showed that D-optimality was maximised with a design using 16 choice tasks (the D-optimality statistics was 97.21%) however with 12 choice tasks the level of D-optimality was still high at 90.86%. Given the challenges encountered when engaging these women with the prior qualitative interviews, we opted for 12 tasks to reduce as much as possible, the respondent burden.

**Sample Size**

Following the approach suggested by Rose and Bliemer, we utilised data from the pilot study to estimate prior coefficients for each attribute (using a conditional logit model without a constant), and combined these with the asymptotic variance-covariance matrix from the experimental design, specifying a significance level of 5% and a t-ratio of 1.96.^4^ The resulting output suggested that with sample sizes >150, it would be possible to estimate significant coefficients for the action, nurse, and cost attributes, but that the location attribute would require a sample size of 1151.

**Statistical Analysis**

As noted in the main paper, because researchers cannot observe an individual’s utility, but do have information on the characteristics / attributes of the alternatives being considered and of the decision makers per se, it is possible to model the statistical relationship between these observed factors and the decision maker’s choices. Of course, there are also factors affecting an individual’s utility, which are not observable to the researcher. Utility can therefore be thought of as having two components as shown below:

| $U_{ni}= V_{ni}+ \varepsilon_{ni}$ | (1) |
| --- | --- |

Where $U_{ni}$ is the utility of the $n^{th}$individual for alternative $i$, $V_{ni}$ is often called the explainable or observable component of utility and is the part of utility determined by observed attributes or participant characteristics, and $\varepsilon_{ni}$ is the unobservable component of utility, thus encompassing other factors affecting utility but not included in $V_{ni}$.^5^ $\varepsilon_{ni}$ can also be thought of as an error term, being the difference between true utility $U_{ni}$ and the part of utility captured by the modelling of $V_{ni}$. As $\varepsilon_{ni}$ for each $i$ is unknown, when modelling, these terms are treated as random, with different models making different assumptions about their distributional form.^5^ As detailed in the main paper, the representative utility ($V$) of the *n^th^* individual for alternative screening intervention ($i$) was estimated to be a linear and additive function of each intervention’s attributes and levels as follows:

| $V_{ni}= \alpha+ \beta_{1}{Action}_{yes}+\beta_{2}{Location}_{GPsurg/clinic}+\beta_{3}{Nurse}_{yes}+\beta_{4}Cost$ | (2) |
| --- | --- |

As specified, equation 2 assumes that the incremental impact of an attribute on utility is fixed or the same for all women, indicating homogeneity of preferences in the study sample. Early discrete choice models included observed heterogeneity by allowing the parameters in equation 2 to vary with participant characteristics (e.g. age, gender, health status or income).^5^ However, preference heterogeneity is expected even in people with the same observed characteristics, and it is important that such unobserved preference heterogeneity is captured in the modelling of discrete choice responses. Besides heterogeneity in the preference parameters, equation 2 is also subject to heterogeneity in the overall variance of the error term.^6^ The variance of the error term includes a scale parameter that can be greater for some participants than for others. This translates into random choice behaviour when participants complete discrete choice tasks. Ignoring these two sources of heterogeneity can lead to potential biases in the estimated parameters and problems with model specification.^7^ It is not possible to estimate separately preference and scale heterogeneity but both sources of heterogeneity can be estimated simultaneously.^8,9^ In this study we estimated a random correlated parameter mixed logit model that has been shown to simultaneously account for the main types of heterogeneity.

We compared the goodness of fit of two models estimated using different sets of parameters as starting values for the random correlated parameter mixed logit model; the first used coefficient values estimated by a conditional multinomial logit model on the same data, and the second used coefficient values from an uncorrelated random parameter mixed logit. Models were estimated using 5000 Halton draws and results compared using the log-likelihood, and Akaike’s Information Criterion divided by the number of observations included in the model (AIC/n). Smaller values on each statistic were taken to indicate a better ‘goodness of fit’.^10^ The best fitting model was the one using parameter estimates from a conditional multinomial logit as starting values and this is the random correlated parameter mixed logit model reported in the main paper (further details are available from the authors upon request).

**References:**

1. Burgess L, Street DJ. Optimal designs for 2(k) choice experiments. *Communications in Statistics-Theory and Methods* 2003; **32**(11): 2185-2206; doi 10.1081/Sta-120024475.

2. Street DJ, Burgess L. Optimal and near-optimal pairs for the estimation of effects in 2-level choice experiments. *Journal of Statistical Planning and Inference* 2004; **118**(1-2): 185-199; doi Pii S0378-3758(02)00399-3

Doi 10.1016/S0378-3758(02)00399-3.

3. Burgess L, Street DJ. Optimal designs for choice experiments with asymmetric attributes. *Journal of Statistical Planning and Inference* 2005; **134**(1): 288-301; doi DOI 10.1016/j.jspi.2004.03.021.

4. Rose JM, Bliemer MCJ. Sample size requirements for stated choice experiments. *Transportation* 2013; **40**(5): 1021-1041; doi 10.1007/s11116-013-9451-z.

5. Train K. *Discrete choice methods with simulation*, 2nd ed. ed. New York, NY : Cambridge University Press: Cambridge, 2009.

6. Swait J, Louviere J. The role of the scale parameter in the estimation and comparison of multinomial logit models. *Journal of Marketing Research* (Peer Reviewed) 1993; **30**(3): 305-314; doi 10.2307/3172883.

7. Hensher D, Louviere J, Swait J. Combining sources of preference data. *Journal of Econometrics* 1999; **89**(1-2): 197-221.

8. Hess S, Train K. Correlation and scale in mixed logit models. *Journal of Choice Modelling* 2017; **23**: 1-8; doi <https://doi.org/10.1016/j.jocm.2017.03.001>.

9. Hess S, Rose JM. Can scale and coefficient heterogeneity be separated in random coefficients models? *Transportation* (journal article) 2012; **39**(6): 1225-1239; doi 10.1007/s11116-012-9394-9.

10. Akaike H. A new look at the statistical model identification. *IEEE Transactions on Automatic Control* 1974; **19**(6): 716-723.

**Figure A1 Final version of the DCE Questionnaire**


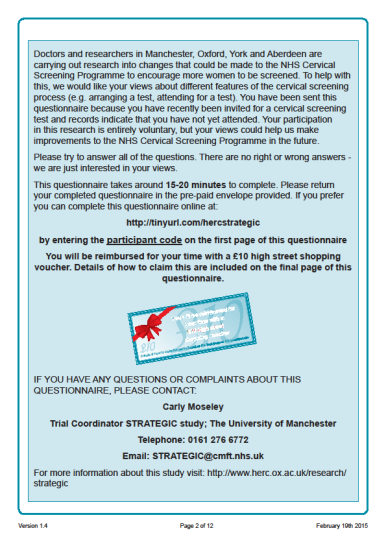

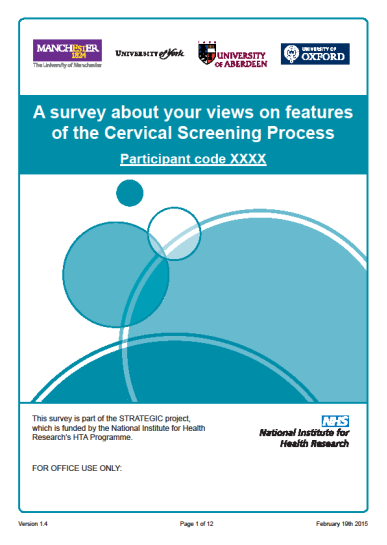


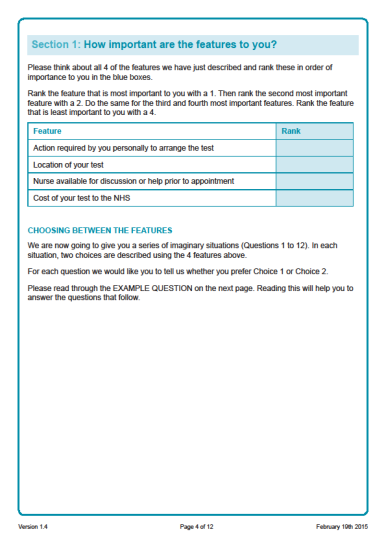

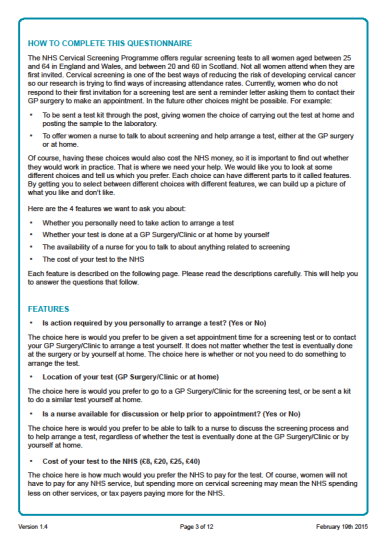


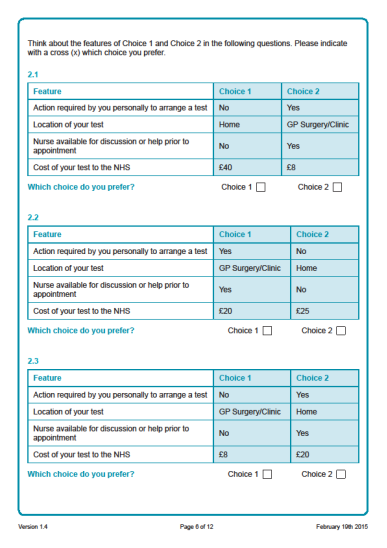

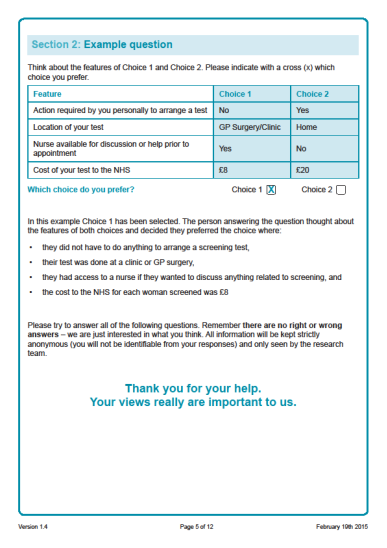


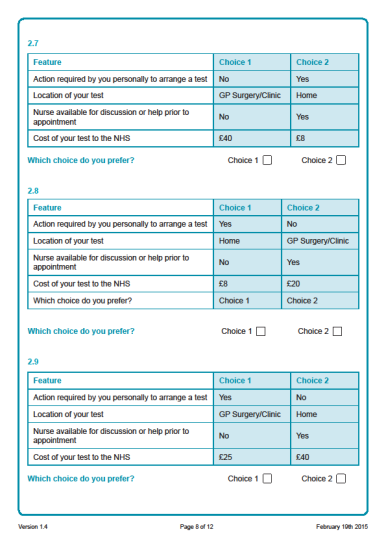

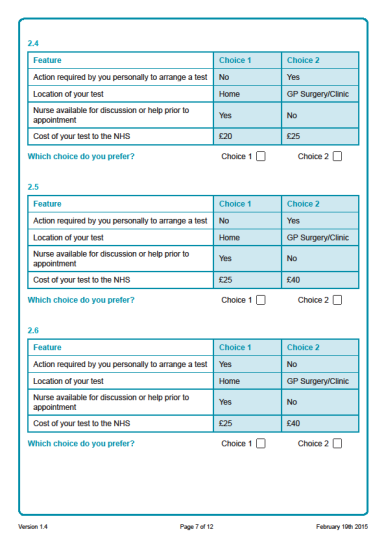


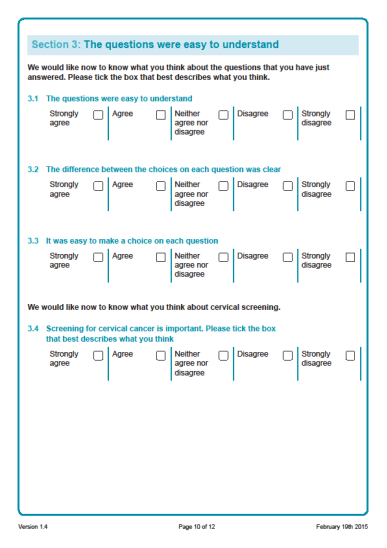

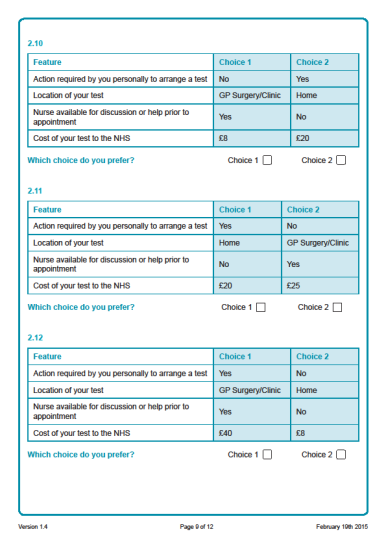


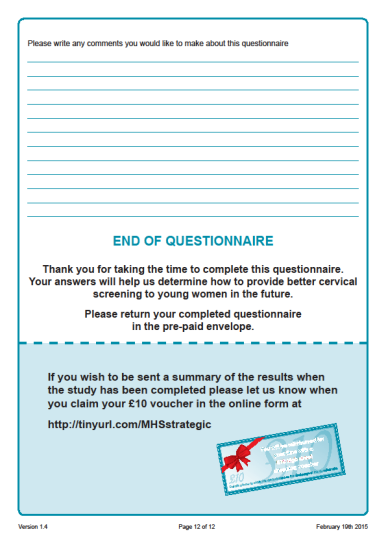

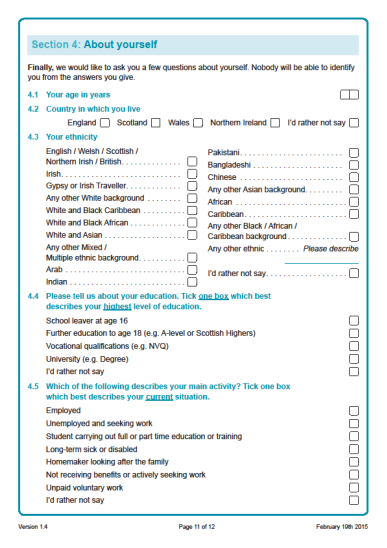


**Figure A2 Patient Information Leaflet**

**
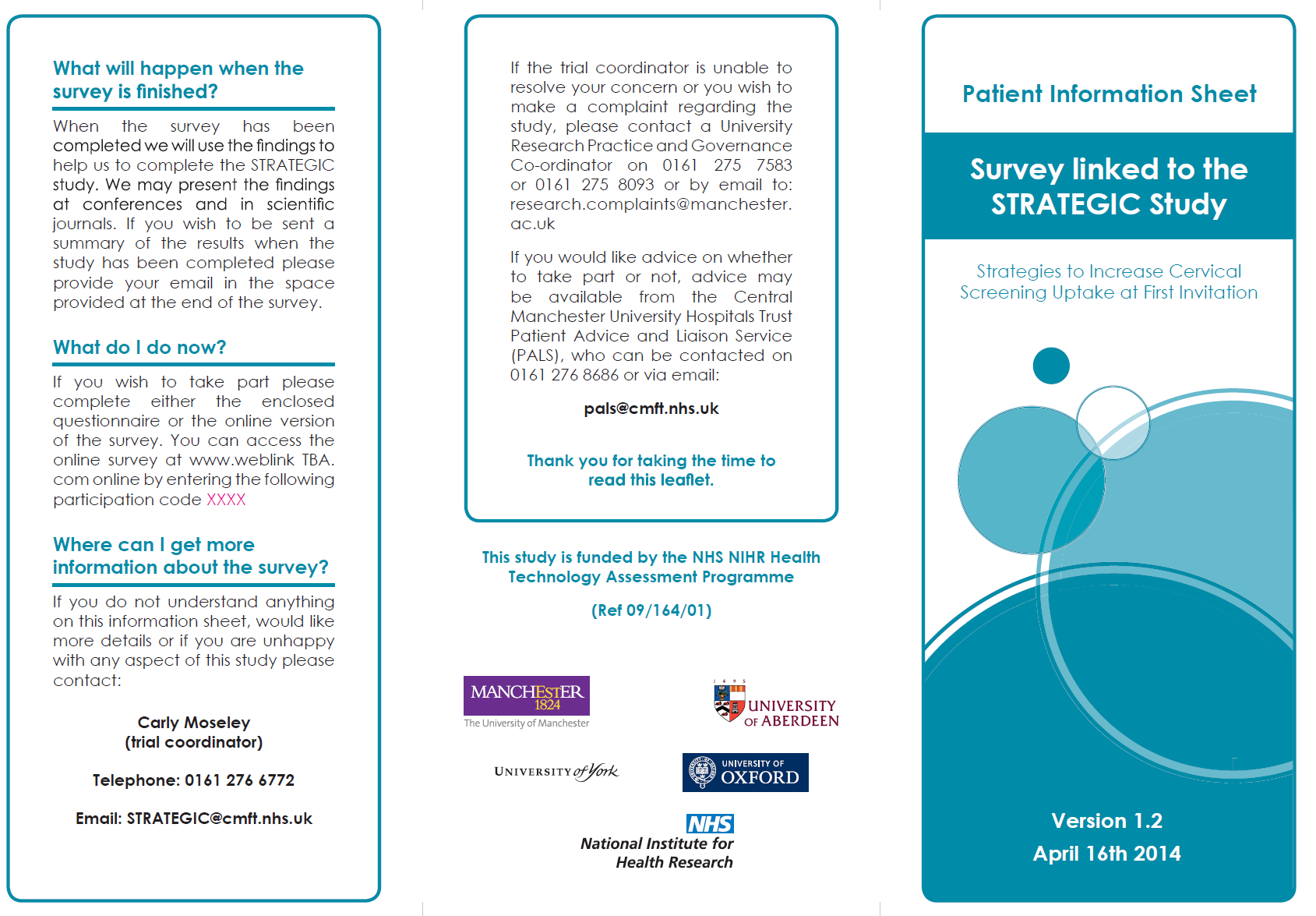
**

**
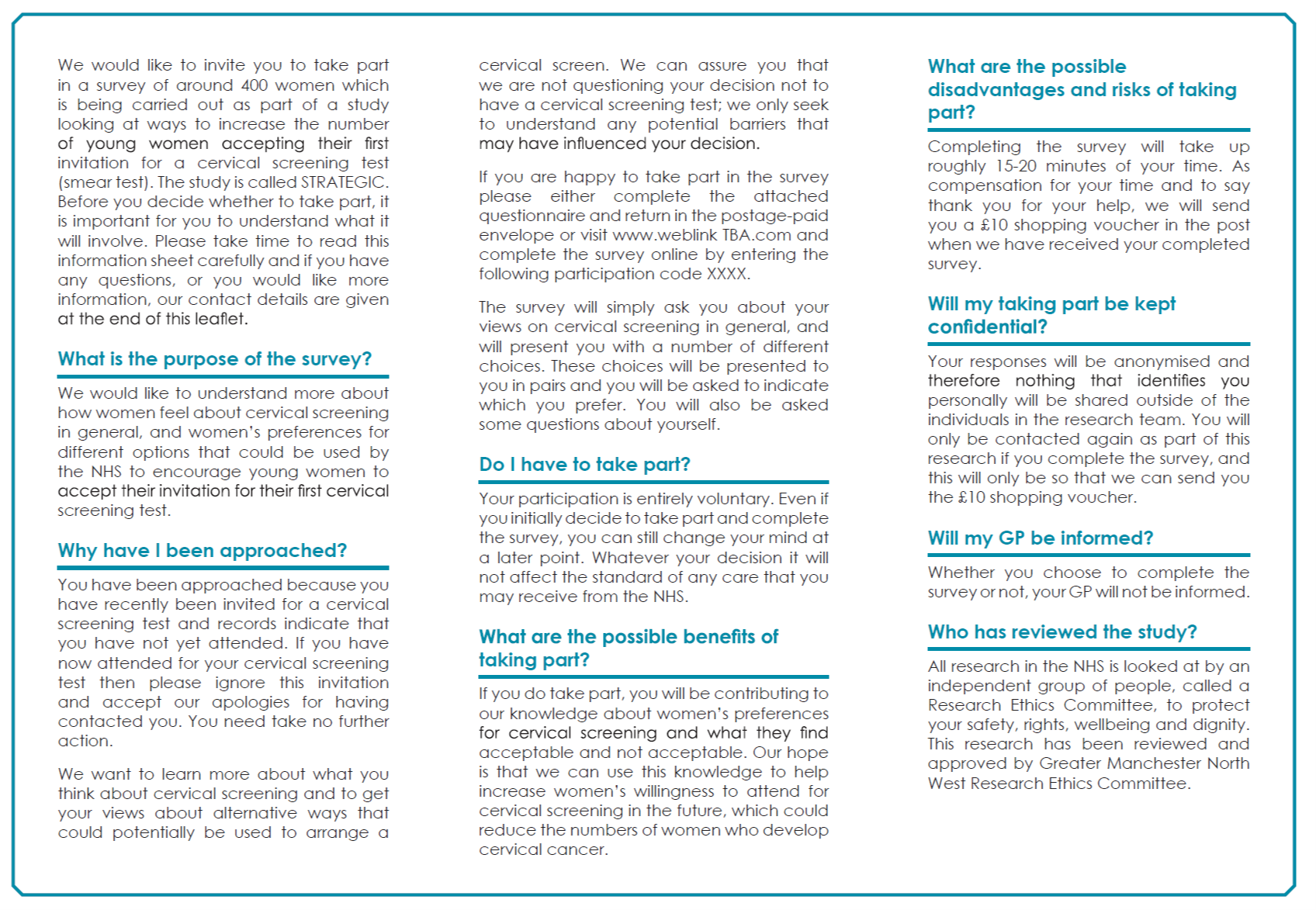
**
